# Antimicrobial Resistance: What Can We Learn from Genomics?

**DOI:** 10.3390/antibiotics14070661

**Published:** 2025-06-30

**Authors:** Tarja Sironen, Ravi Kant

**Affiliations:** 1 Department of Virology, Medicum, University of Helsinki, 00290 Helsinki, Finland; tarja.sironen@helsinki.fi; 2 Department of Basic Veterinary Sciences, Faculty of Veterinary Medicine, University of Helsinki, 00790 Helsinki, Finland; 3 Department of Tropical Parasitology, Institute of Maritime and Tropical Medicine, Medical University of Gdansk, 81-519 Gdynia, Poland

## 1. Antimicrobial Resistance and Genomics: Illuminating New Frontiers in a Global Crisis

Antimicrobial resistance (AMR) has rapidly emerged as one of the most pressing threats to global health, development, and sustainability. Fueled by the mis- and overuse of antibiotics, insufficient infection control, and gaps in water and sanitation infrastructure, the rise in drug-resistant pathogens not only endangers human and animal health but also threatens economic stability and social progress worldwide. Without coordinated action, AMR could undermine the very foundation of modern medicine and derail efforts to meet the United Nations Sustainable Development Goals [1,2,3].

Against this backdrop, genomics has become an indispensable tool in our arsenal. Advances in high-throughput sequencing technologies have revolutionized how we detect, monitor, and understand antimicrobial resistance. It is now possible to rapidly decode the genomes of pathogenic microbes with unprecedented accuracy and speed, offering insights into resistance mechanisms, virulence factors, transmission dynamics, and outbreak source tracing. These developments are transforming diagnostics, surveillance, and therapeutic strategies across the globe [3,4,5,6].

This Special Issue brings together groundbreaking research that harnesses genomic, metagenomic, and comparative genomic approaches to uncover critical dimensions of AMR. By characterizing resistance genes, mobile genetic elements, and clonal lineages, these studies offer a deeper understanding of how resistance emerges, spreads, and persists across diverse ecological and geographical contexts.

Below is an overview of the six manuscripts featured in this collection:

- **Bacillus cereus Group Diversity and AMR in Foodstuffs**: Sornchuer et al. [7] used whole-genome sequencing to investigate *B*. *cereus* isolates from food in Thailand. Their work highlights the co-occurrence of virulence and resistance genes in both pathogenic and non-pathogenic strains, raising food safety and public health concerns.
- **One Health AMR Surveillance in *E. coli***: Jewell et al. [8] leveraged WGS to assess the AMR gene distribution in *E. coli* across humans, animals, food, and environmental sources in Washington State. Their study underscores the feasibility and power of genomic surveillance within a One Health framework.
- **Multidrug-Resistant E. coli ST410 in Egypt**: Mohamed et al. [9] characterized a high-risk *E. coli* clone co-harboring ESBL and carbapenemase genes, including *blaNDM-5*. The discovery of chromosomal integration of *blaCMY-2* emphasizes the urgent need for surveillance in clinical settings.
- **Methicillin-Resistant *Staphylococcus epidermidis***: Altayb et al. [10] reported on the genomic features of multidrug-resistant *S. epidermidis*, identifying biofilm-associated genes and unique SCCmec elements that complicate treatment options for nosocomial infections.
- **Comparative Genomics of *Arcanobacterium phocae* Strains:** Aaltonen et al. [11] conducted whole-genome sequencing of 42 *A. phocae* strains isolated from seals and various fur animals. Their findings reveal distinct phylogenetic clusters between marine and terrestrial hosts, alongside virulence-associated proteins of interest for vaccine development, highlighting the need for targeted prevention strategies in the fur industry.
- **Pan and Core Genome Analysis of *Mycobacterium tuberculosis* Strains:** Zakham et al. [12] performed a comparative genome analysis of 183 *M. tuberculosis* strains, including BCG vaccine variants. The study revealed high inter-species diversity and identified conserved virulence genes within the core genome, offering valuable insights for future TB vaccine development and the assessment of attenuated strain safety.

These articles collectively demonstrate how genomic insights can guide evidence-based interventions and policies to combat AMR. They emphasize the urgency of cross-sectoral collaboration, robust surveillance systems, and sustainable stewardship of antimicrobial agents.

## 2. Future Directions

As AMR continues to evolve, several key areas require further exploration:

- **Strengthening the One Health Approach**: Understanding the interconnectedness of human, animal, and environmental health is vital for predicting and preventing AMR emergence.
- **Global Genomic Surveillance**: Expanding genomic monitoring networks will enable early detection of high-risk clones and resistance genes before they become entrenched in clinical settings.
- **Bioinformatics and Predictive Tools**: Developing advanced computational platforms for real-time analysis and prediction of resistance evolution will support faster public health responses.
- **Novel Therapeutics and Alternatives**: Investment in the development of new antimicrobials, bacteriophage therapy, and microbiome modulation could offer viable alternatives to current treatments.
- **Public Health Policy Integration**: Genomic data must inform global policy decisions, antimicrobial stewardship programs, and infection prevention strategies.

By embracing the transformative potential of genomics, we can better understand and mitigate the global AMR crisis. This Special Issue not only showcases exemplary research but also calls for continued innovation and collaboration to secure the future of effective infectious disease management.
